# Supplementary material for: Cancer treatment monitoring using cell-free DNA fragmentomes
Source: Nat Commun. 2024 Oct 21;15:8801. doi: 10.1038/s41467-024-53017-7 (PMC11493959; doi:10.1038/s41467-024-53017-7)
Supplement: Supplementary file 1 — Supplementary Information [file 41467_2024_53017_MOESM1_ESM.pdf]

Cancer treatment monitoring using cell-free DNA fragmentomes

Supplementary Information

Iris van 't Erve[1*], Bahar Alipanahi[2*], Keith Lumbard[2*], Zachary L. Skidmore[2*], Lorenzo Rinaldi[2], Laurel K. Millberg[2], Jacob Carey[2], Bryan Chesnick[2], Stephen Cristiano[2], Carter Portwood[2], Tony Wu[2], Erica Peters[2], Karen Bolhuis[3], Cornelis J. A. Punt[4], Jennifer Tom[2], Peter B. Bach[2], Nicholas C. Dracopoli[2], Gerrit A. Meijer[1], Robert B. Scharpf[5], Victor E. Velculescu[5], Remond J.A. Fijneman[1#], Alessandro Leal[2,6#]

[1]Department of Pathology, Netherlands Cancer Institute, Amsterdam, The Netherlands
[2]Delfi Diagnostics, Inc., Baltimore, MD, United States
[3]Department of Medical Oncology, Amsterdam UMC, Cancer Center Amsterdam, University of Amsterdam, the Netherlands
[4]Julius Center for Health Sciences and Primary Care,
University Medical Center Utrecht, Utrecht, The Netherlands
[5]The Sidney Kimmel Comprehensive Cancer Center,
Johns Hopkins University School of Medicine, Baltimore, MD, United States
[6]NYU Langone Health Perlmutter Comprehensive Cancer Center, New York, NY, United States

*These authors contributed equally
[#]To whom correspondence should be addressed:
Remond Fijneman (r.fijneman@nki.nl) or
Alessandro Leal (alessandro.leal@nyulangone.org)

Supplementary Figures

Supplementary Fig. 1. – CAIRO5 study design

Supplementary Fig. 2. – Patient flow diagram

Supplementary Fig. 3. – Plasma cfDNA fragmentation profiles of CRC patients are related to structure of CRC

Supplementary Fig. 4. – cfDNA tumor fraction assessed by MAF, DELFI-TF, and ichorCNA

Supplementary Fig. 5. – DELFI-TF captures cancer signals in copy number neutral and altered regions

Supplementary Fig. 6. – DELFI-TF and MAF measurements reflect imaging assessments at baseline

Supplementary Fig. 7. – DELFI-TF and MAFs but not CEA predict progression-free survival in colorectal cancer patients

Supplementary Fig. 8. – DELFI-TF and colorectal cancer clinical outcomes

Supplementary Fig. 9. – DELFI-TF and MAF dynamics in CAIRO5 patients in the mutant arm

Supplementary Fig. 10. – DELFI-TF dynamics in CAIRO5 patients in the wild type arm

Supplementary Fig. 11. – Imaging and plasma biomarkers for survival outcomes in metastatic colorectal cancer patients

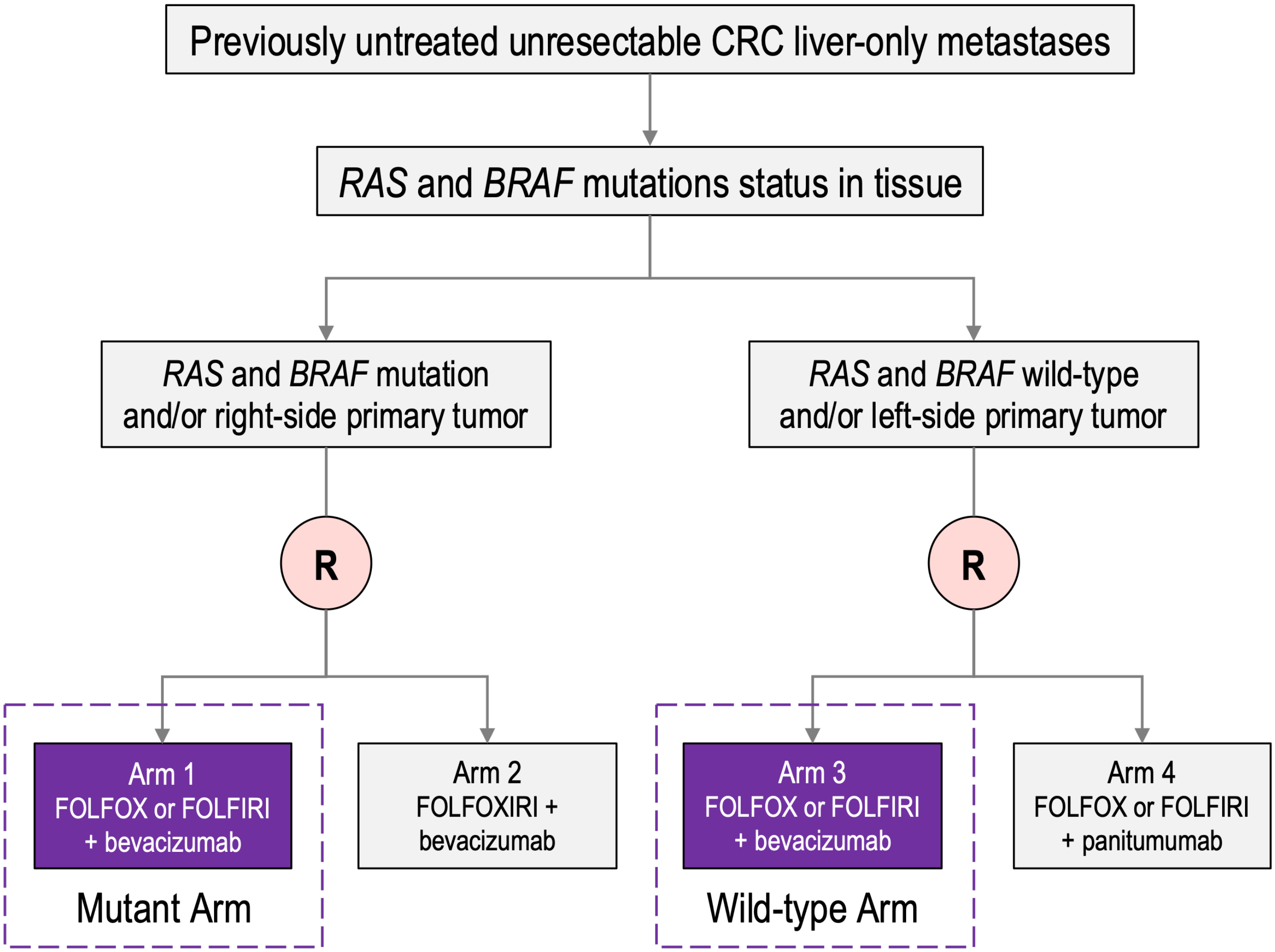

Supplementary Fig. 1. CAIRO5 study design. Once eligibility was confirmed, including the unresectable status of liver metastases as defined by the central panel, *KRAS* (exon 2, 3, and 4), *NRAS* (exon 2 and 3), and *BRAF V600* mutation status were assessed in tissue samples. Patients with *RAS/BRAF* mutant tumors were randomized between doublet chemotherapy plus bevacizumab (Arm A) or triple chemotherapy plus bevacizumab (Arm B). Patients with *RAS/BRAF* wildtype tumors were randomized between doublet chemotherapy plus either bevacizumab (Arm C) or panitumumab (Arm D). In the present translational study, blood draws from patients in Group A (*RAS/BRAF* mutant) and Group C (*RAS/BRAF* wildtype) were processed and analyzed.

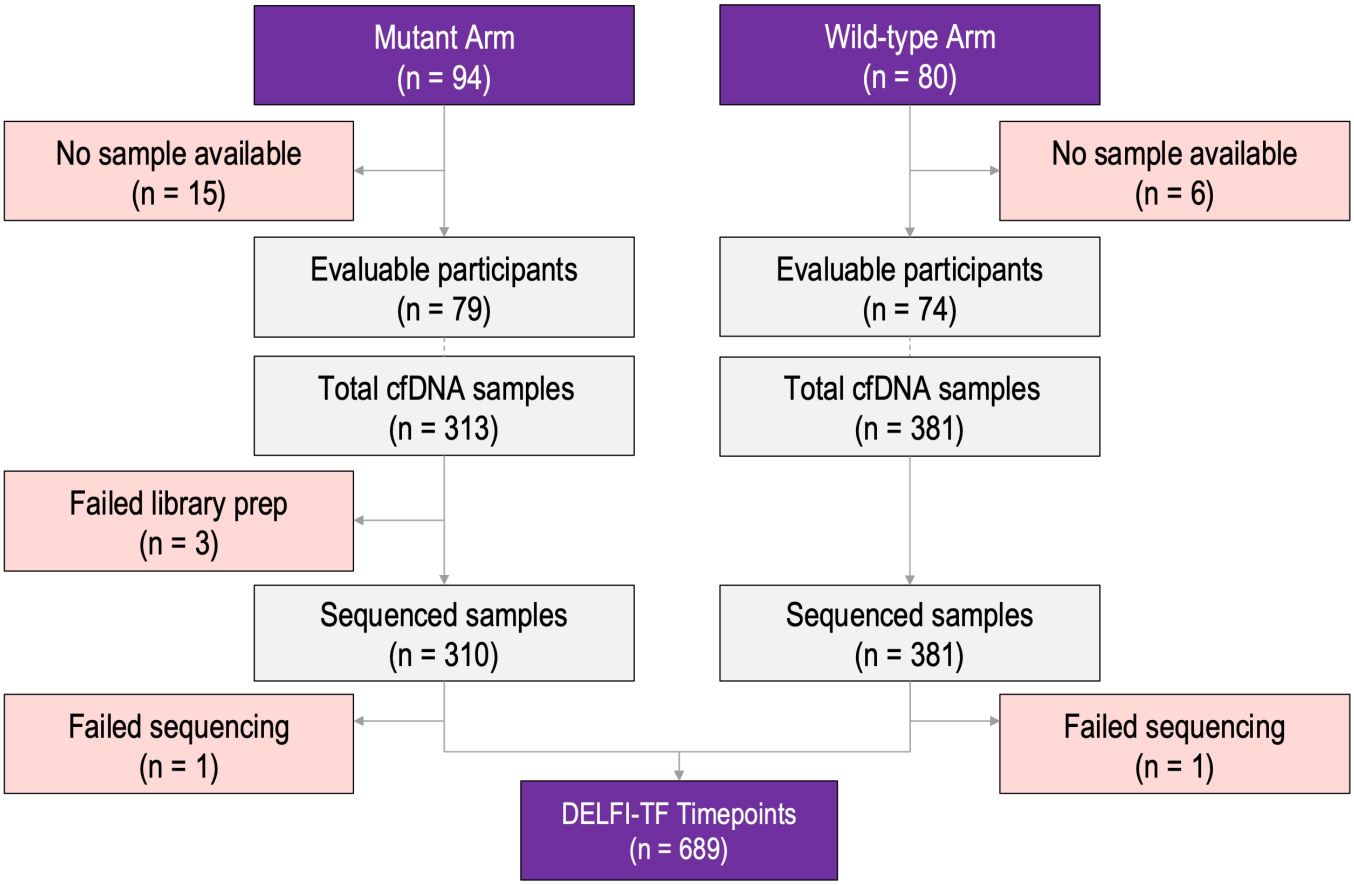

Supplementary Fig. 2. Patient flow diagram. The number of patients and samples included in the study and the reasons for exclusion are depicted. In total, 153 patients were analyzed in two study groups: patients with a *RAS/BRAF* mutant tumor (n = 79) and patients with a *RAS/BRAF* wildtype tumor (n = 74). Only 5 out of 694 processed samples failed to produce final high-quality sequencing data. DELFI-TF values were calculated for 689 longitudinal samples.

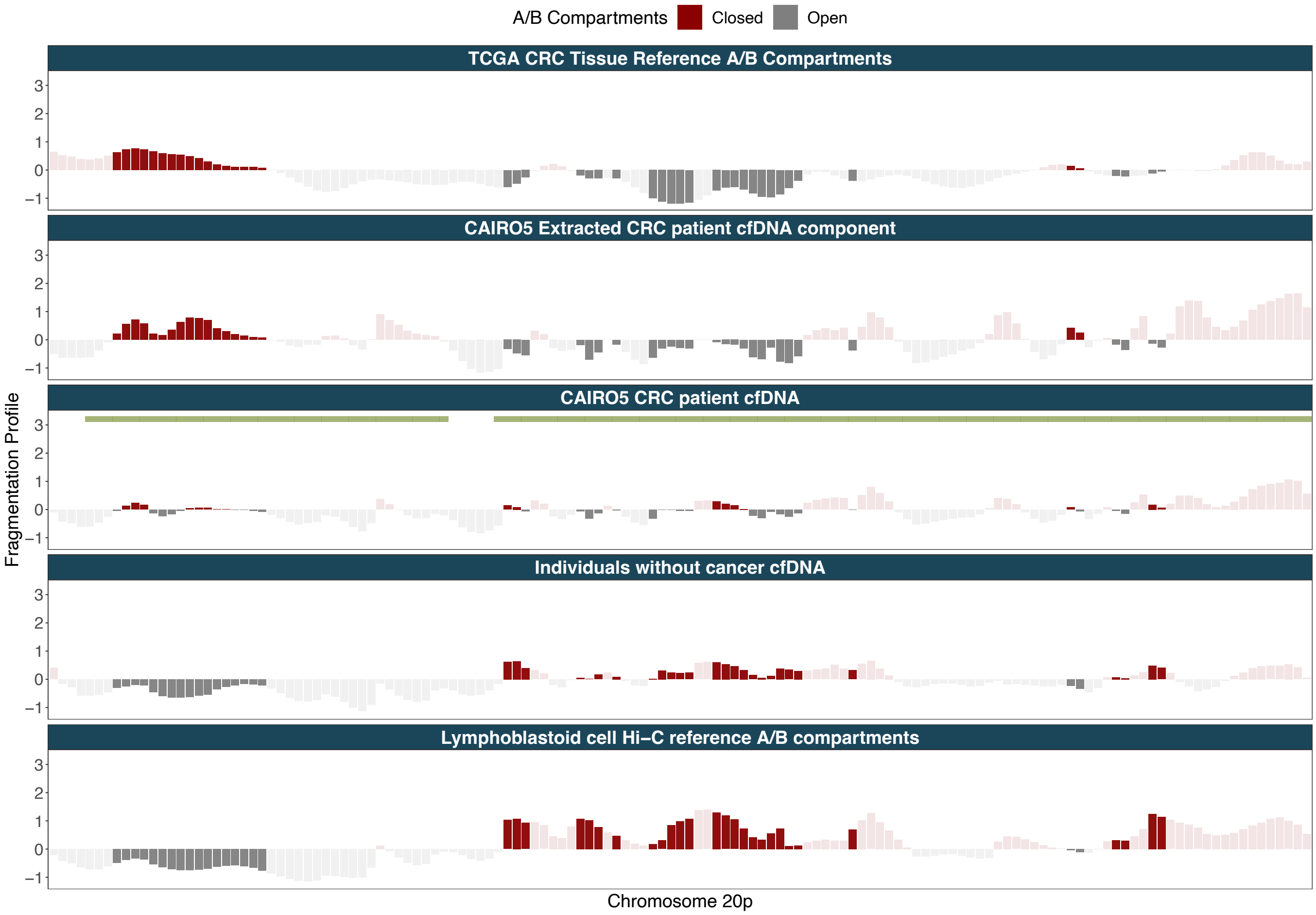

Supplementary Fig 3. Plasma cfDNA fragmentation profiles of CRC patients are related to chromatin structure of CRC. Comparison of open and closed A/B compartments across disease and healthy samples in chromosome 20. Starting from top: A/B compartments from TCGA colon adenocarcinoma reference tissue; Median CRC component extracted from 10 patient samples with the highest DELFI-TF scores; Median fragmentation profile for the aforementioned CRC samples in plasma; Median fragmentation profile for 10 non-cancer samples in plasma; A/B compartments for a lymphoblastoid cell line. Green bars above bins represent coordinates in the genome which are copy neutral for 5 or more of the 10 CRC samples evaluated.

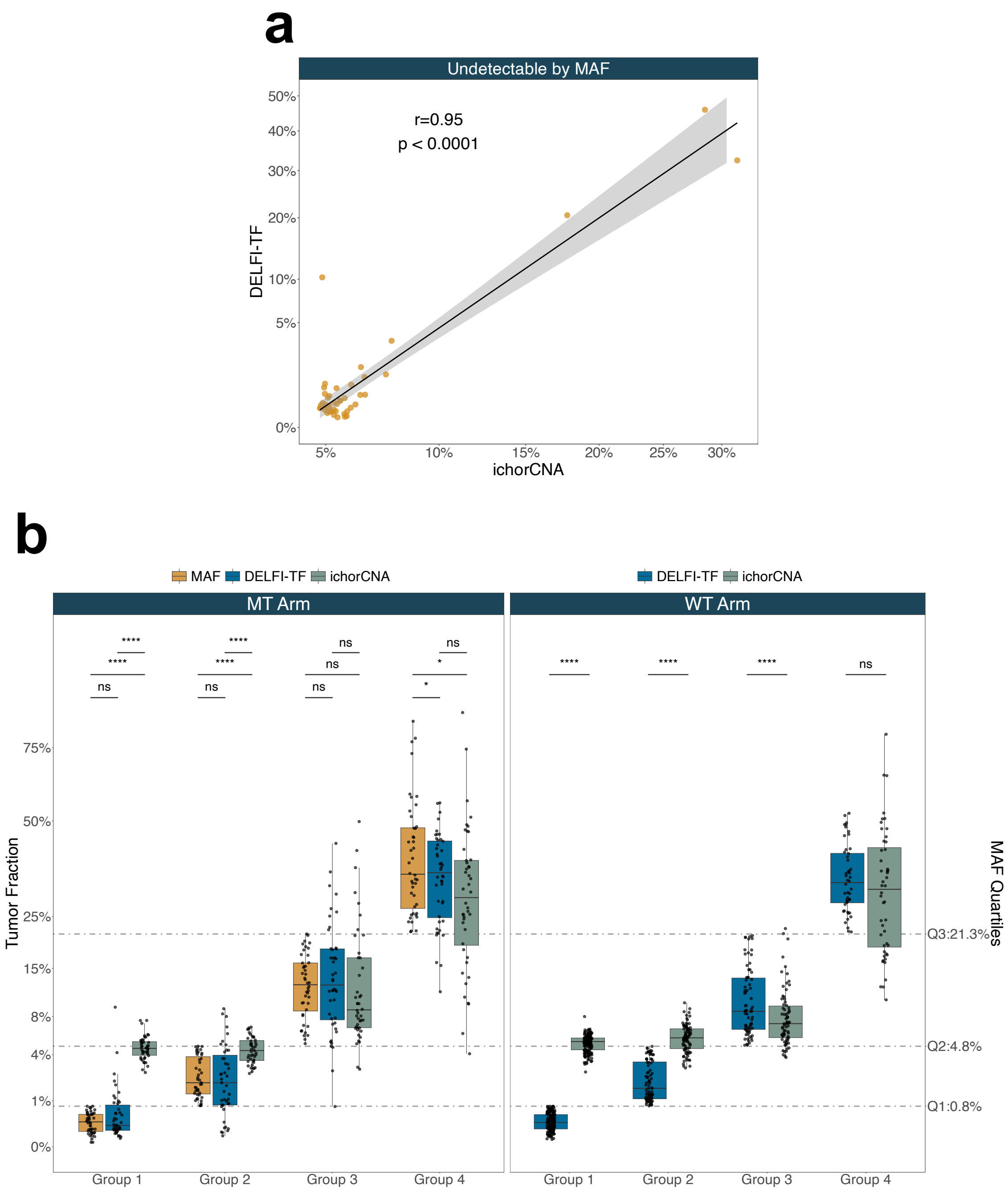

Supplementary Fig. 4. cfDNA tumor fraction assessed by MAF, DELFI-TF and ichorCNA. a, DELFI-TF and ichorCNA correlation for samples (n=45) with undetectable MAF and ichorCNA above Q2 threshold in panel A (4.8%). High correlation of DELFI-TF and ichorCNA values was observed, strongly supporting the notion that these patients did indeed have detectable ctDNA that was missed with ddPCR (r=0.95, *p*=3.77e-24, two-sided Pearson correlation). **b,** DELFI-TF, MAF and ichorCNA values for all samples belonging to the mutant arm (left) and the wild type arm (right). Samples within the mutant arm are divided into four groups corresponding to MAF quartiles (Q1-Q4): Group 1 (n=46) includes all samples less than or equal to Q1 but greater than 0, Group 2 (n=43) includes all samples greater than Q1 and less than or equal to Q2 , Group 3 (n=44) includes all samples greater than Q2 and less than or equal to Q3, Group 4 (n=44) includes all samples greater than Q3. Samples in the wild type arm are subset on DELFI-TF using ctDNA thresholds identified in the MT arm from ddPCR (Group 1 n=169; Group 2 n=88; Group 3 n=74; Group 4 n=48). Overall, DELFI-TF demonstrates greater concordance with MAF in comparison to ichorCNA (left) (Paired two-sided Wilcoxon Rank-Sum, Bonferroni Correction) and the disparity between DELFI-TF and ichorCNA is retained in the wild type arm within groups (left/right). Ribbons around the regression line in correlation plots represent the 95% confidence level interval for predictions.

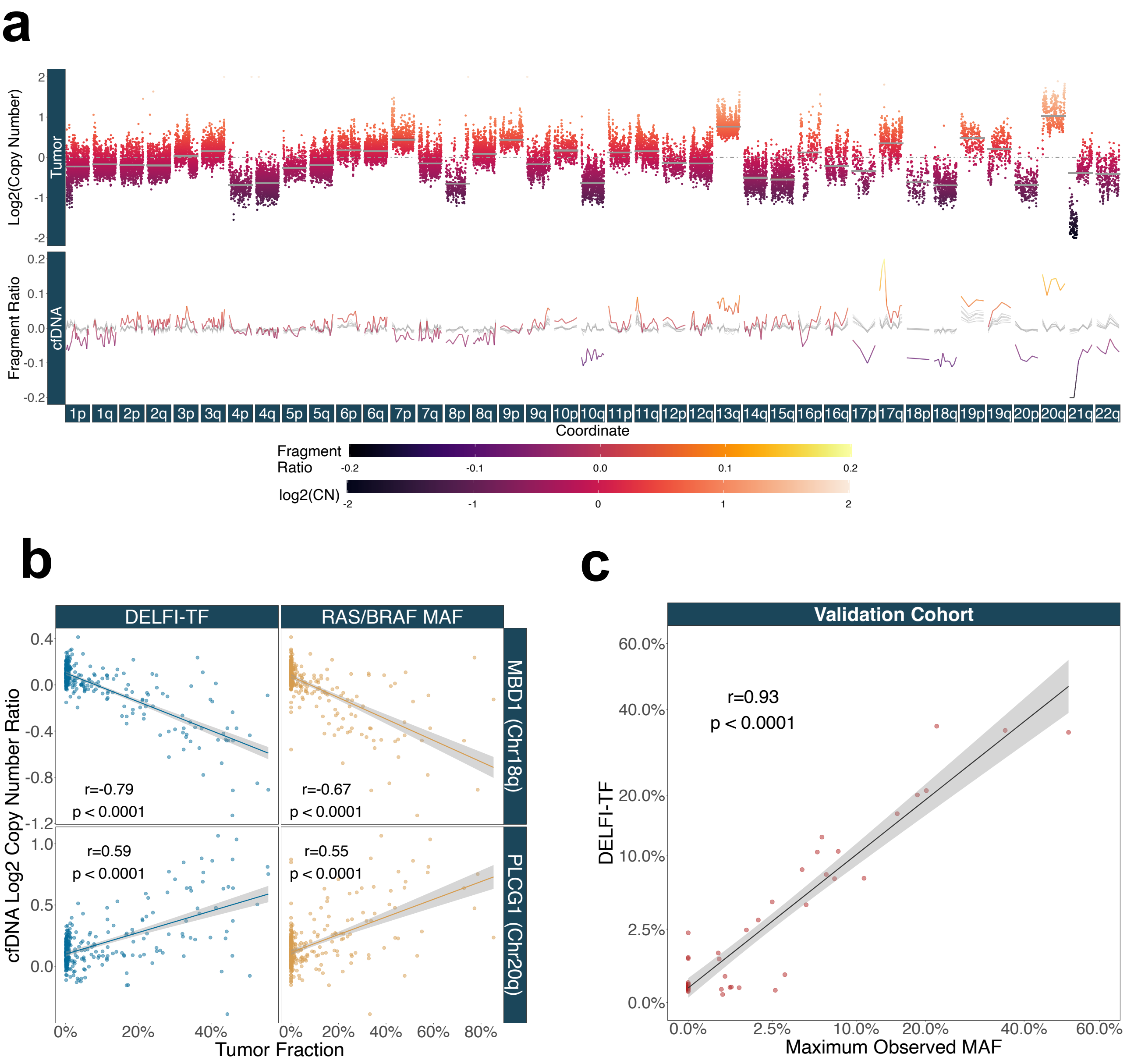

Supplementary Fig. 5. DELFI-TF captures cancer signals in copy number neutral and altered genomic regions. a, cfDNA fragmentation profiles (bottom) in a patient with wild type mCRC, plotted alongside 10 non-cancer individuals (gray lines), exhibit short-to-long ratio aberrations even in the context of tumor copy neutral regions across 100-kb bins in a matched tissue sample (top). **b**, Plasma tumor fractions assessed by DELFI-TF (blue) and RAS/BRAF mutation allele frequency (MAF; gold) values correlate with cfDNA copy number changes in genomic regions that harbor frequently deleted (MBD1, n=309; MAF, r=-0.67 *p*=2.50e-41; DELFI-TF, r=-0.79, *p*=4.54e-68; two-sided Pearson correlation) or amplified (PLCG1, n=309; MAF, r=0.55 *p*=1.55e-25; DELFI-TF, r=0.59, *p*=3.35e-30; two-sided Pearson correlation) genes in colorectal cancer.**c,** DELFI-TF predictions correlate with the max MAF from a targeted sequencing assay in an independent validation cohort (n=47, r=0.93, *p*=2.20e-16, two-sided Pearson correlation). All mutations identified as non-hotspots and germline were removed from this analysis. Ribbons around the regression line in correlation plots represent the 95% confidence level interval for predictions.

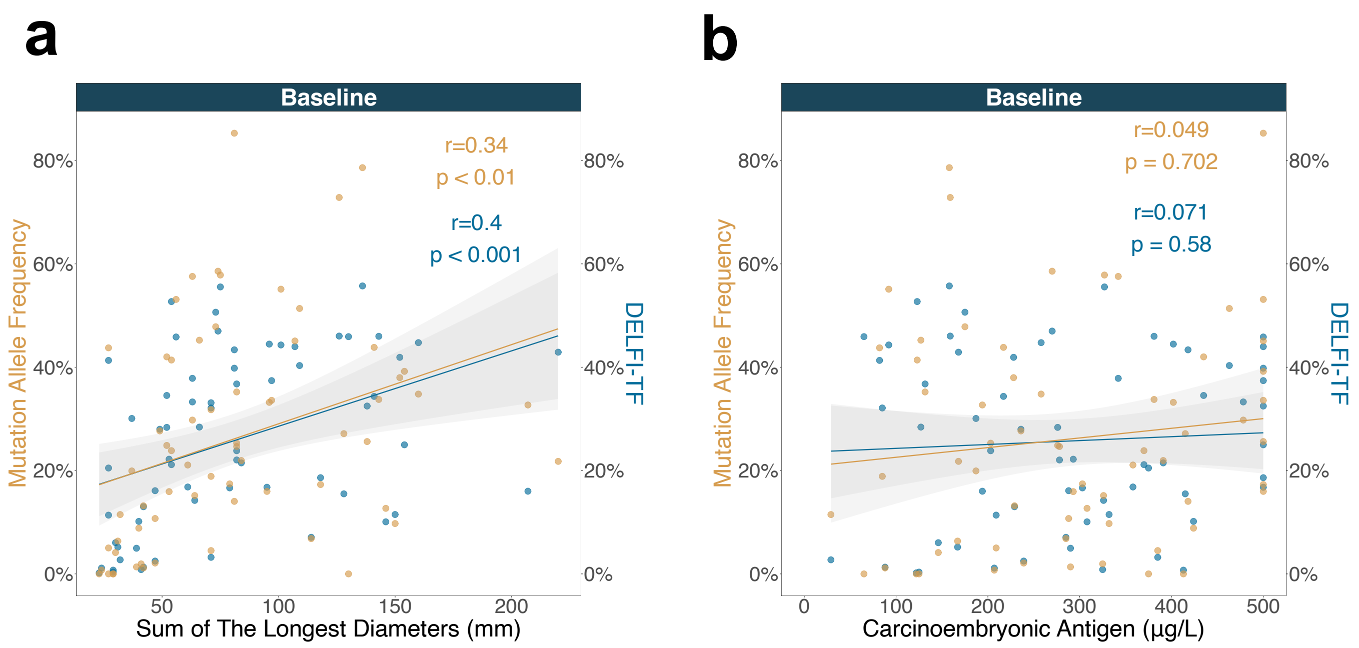

Supplementary Fig. 6. DELFI-TF and MAF measurements reflect imaging assessments at baseline. a, DELFI-TF (blue) and *RAS/BRAF* MAF (gold) values at baseline exhibit a moderate correlation with the Sum of Longest Diameters (SLDs) of liver target lesions in pre-treatment imaging scans (DELFI-TF, n=65, *r* = 0.40, *p=9.04e-04*, two-sided Pearson Correlation; MAF, n=65, *r* = 0.34, *p=5.78e-03*, two-sided Pearson Correlation). **b**, DELFI-TF (blue) and *RAS/BRAF* MAF (gold) values at baseline exhibit no significant correlation with baseline levels of carcinoembryonic antigen (DELFI-TF, n=64, *r* = 0.07, *p=5.80e-1*, Pearson Correlation; MAF, n=64, *r* = 0.05, *p=7.02e-1*, Pearson Correlation). Ribbons around the regression line in correlation plots represent the 95% confidence level interval for predictions.

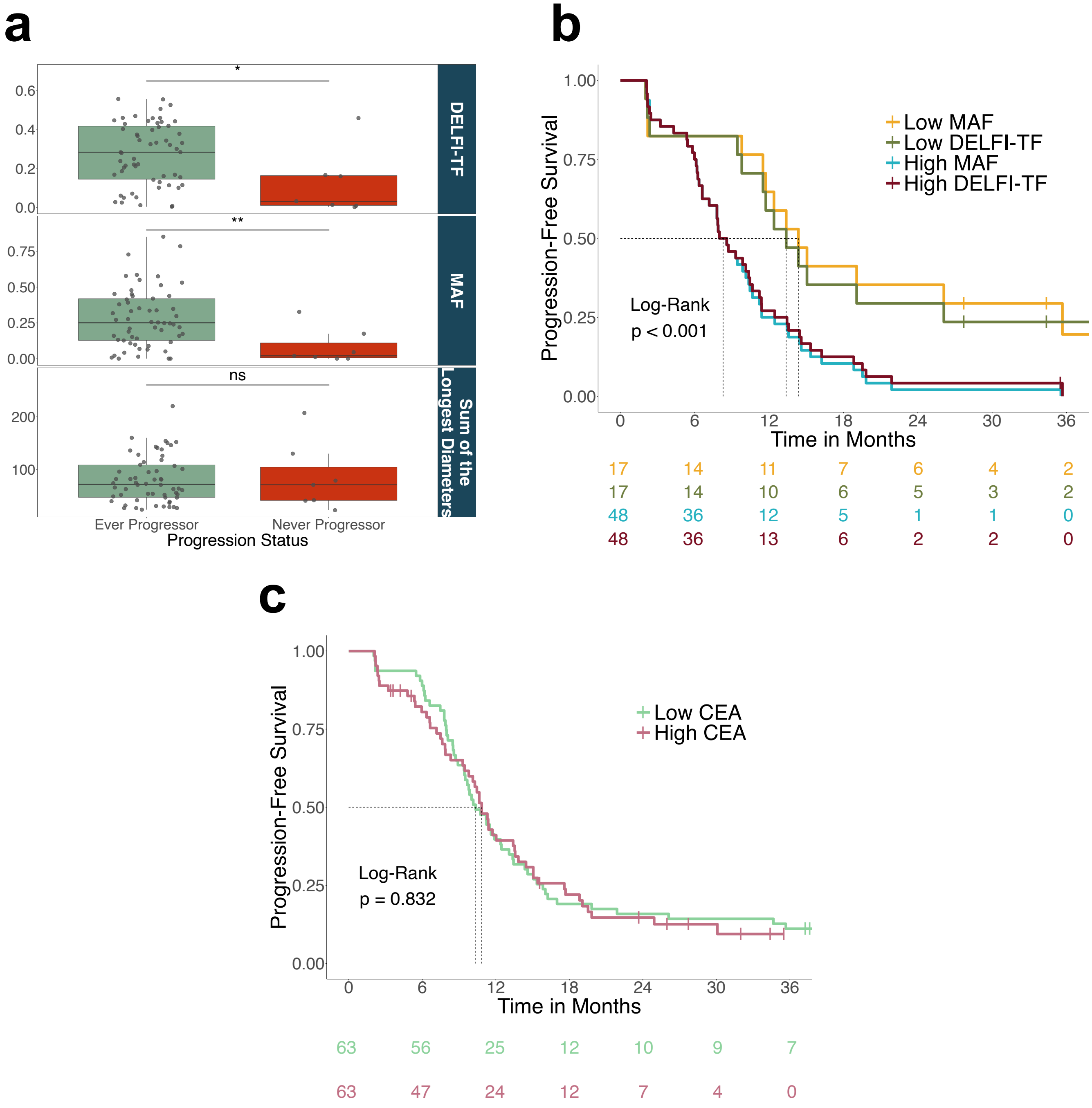

Supplementary Fig. 7. DELFI-TF and MAFs but not CEA predict progression-free survival in colorectal cancer patients. a, Tumor fractions assessed by DELFI-TF and MAF at baseline exhibit a higher trend in patients who eventually experienced disease progression (green, n=58) than patients who never experienced disease progression (red, n=7) during the study follow-up (DELFI-TF, *p=2.17e-2*, two-sided Wilcoxon Rank-Sum; MAF, *p*=6.96e-3, two-sided Wilcoxon Rank-Sum). The sum of the longest diameters at baseline did not differ between ever progressors (green, n=58) and never progressors (red, n=7) (*p*=7.83e-1, two-sided Wilcoxon Rank-Sum). **b**, Kaplan-Meier curves for Progression-Free Survival (PFS) according to baseline DELFI-TF and MAF values. Patients with low DELFI-TF (green) or low MAF (yellow) experienced significantly longer PFS than patients with high DELFI-TF (red) or high MAF (blue) (*p=5.26e-04*, Log-Rank). Low/High DELFI-TF and MAF values were categorized as below or above the 25th percentile distribution of tumor fraction among the population of *RAS/BRAF* mutant individuals (n = 65). **c**, Kaplan-Meier curves for PFS according to baseline carcinoembryonic antigen (CEA) values below (green) or above (red) the median among patients with metastatic colorectal cancer (n = 126) (*p=8.32e-1*, Log-Rank). The lower whisker is similarly constructed using the 25th percentile.

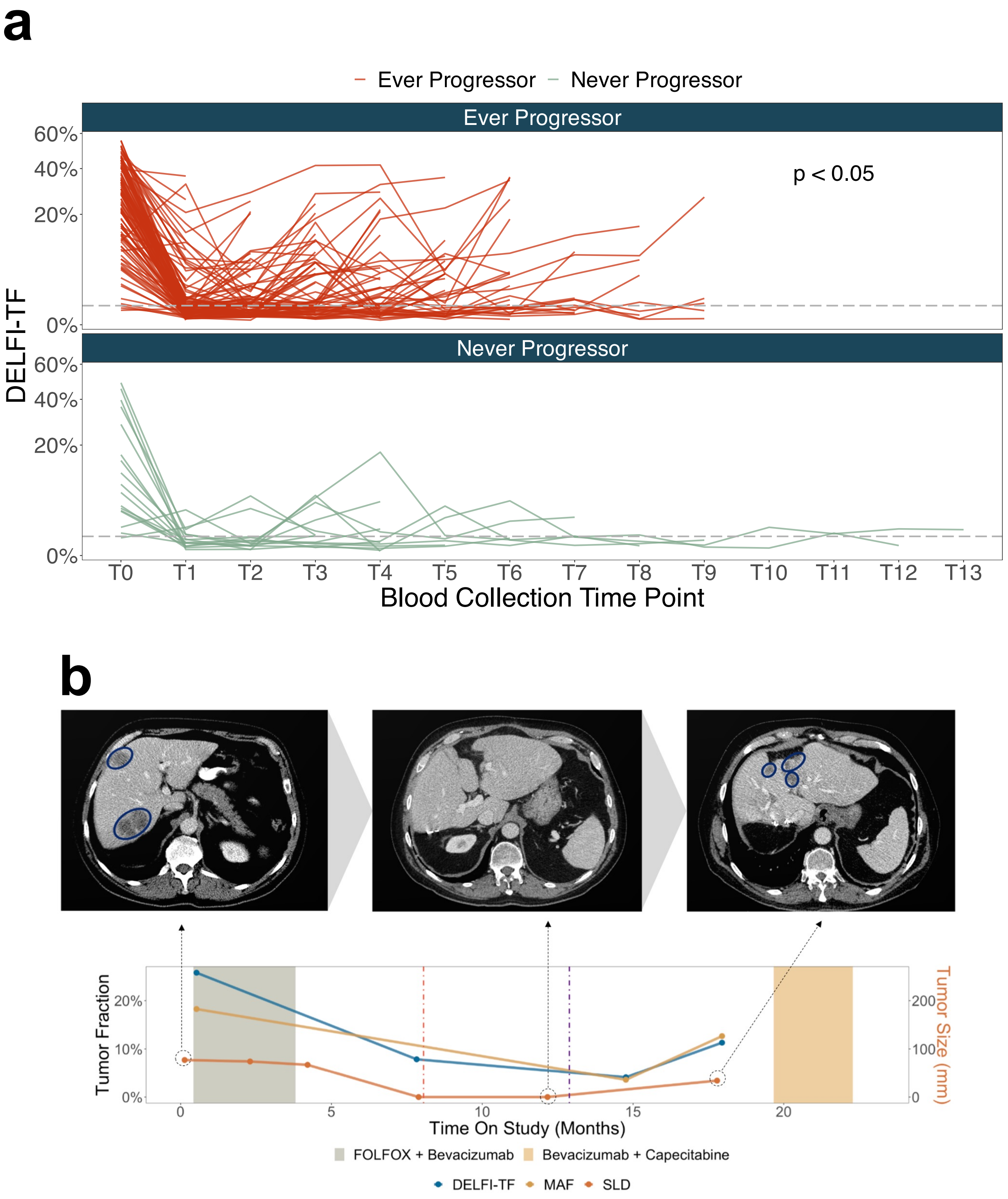

Supplementary Fig. 8. DELFI-TF and colorectal cancer clinical outcomes. **a,** Patients who eventually experienced disease progression (top, n=109) more often exhibited significantly increased DELFI-TF values at longitudinal time points than patients who never presented with disease progression (bottom,n=17) (Median Never vs Ever Progressor T1-Tn, *p=4.56e-2*, two-sided Wilcoxon Rank-Sum). **b**, Liver metastases are highlighted by blue circles in longitudinal imaging scans (top). cfDNA tumor fraction dynamics (DELFI-TF, MAF) and the SLD values are shown for study patient 11 (bottom). Treatments are indicated by shaded bars. The purple dotted line represents the time for primary tumor resection a few weeks after liver metastases removal. DELFI-TF and ddPCR MAF for the *KRAS* G12D mutation accurately track disease burden dynamics before and after the complete resection.

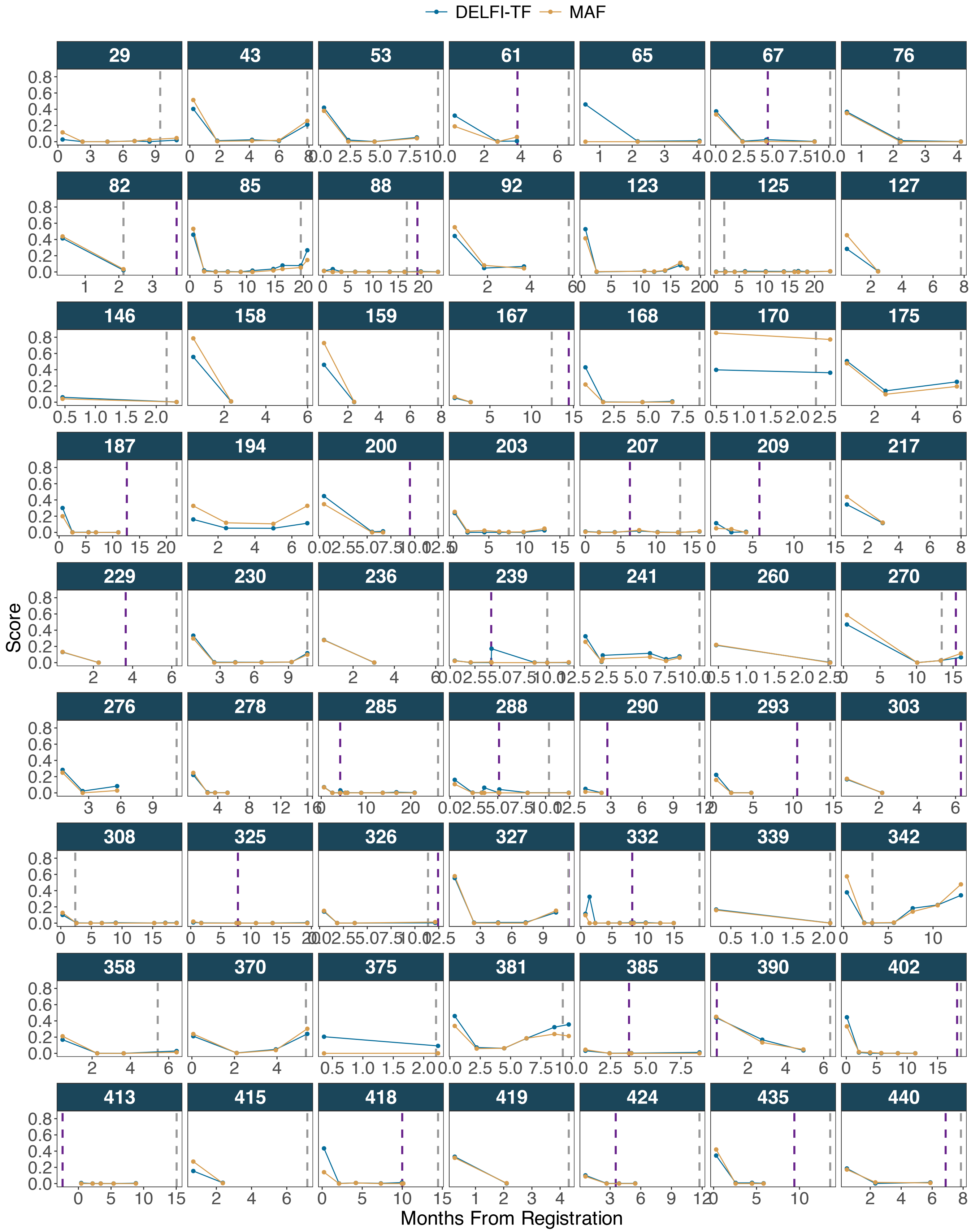

Supplementary Fig. 9. DELFI-TF and MAF dynamics in CAIRO5 patients in the mutant arm. Longitudinal DELFI-TF mimics ddPCR MAF dynamics in patients with *RAS/BRAF* driver mutations. Purple vertical dashed lines represent surgical time points. Gray vertical dashed lines represent disease progression documentation by RECIST1.1.

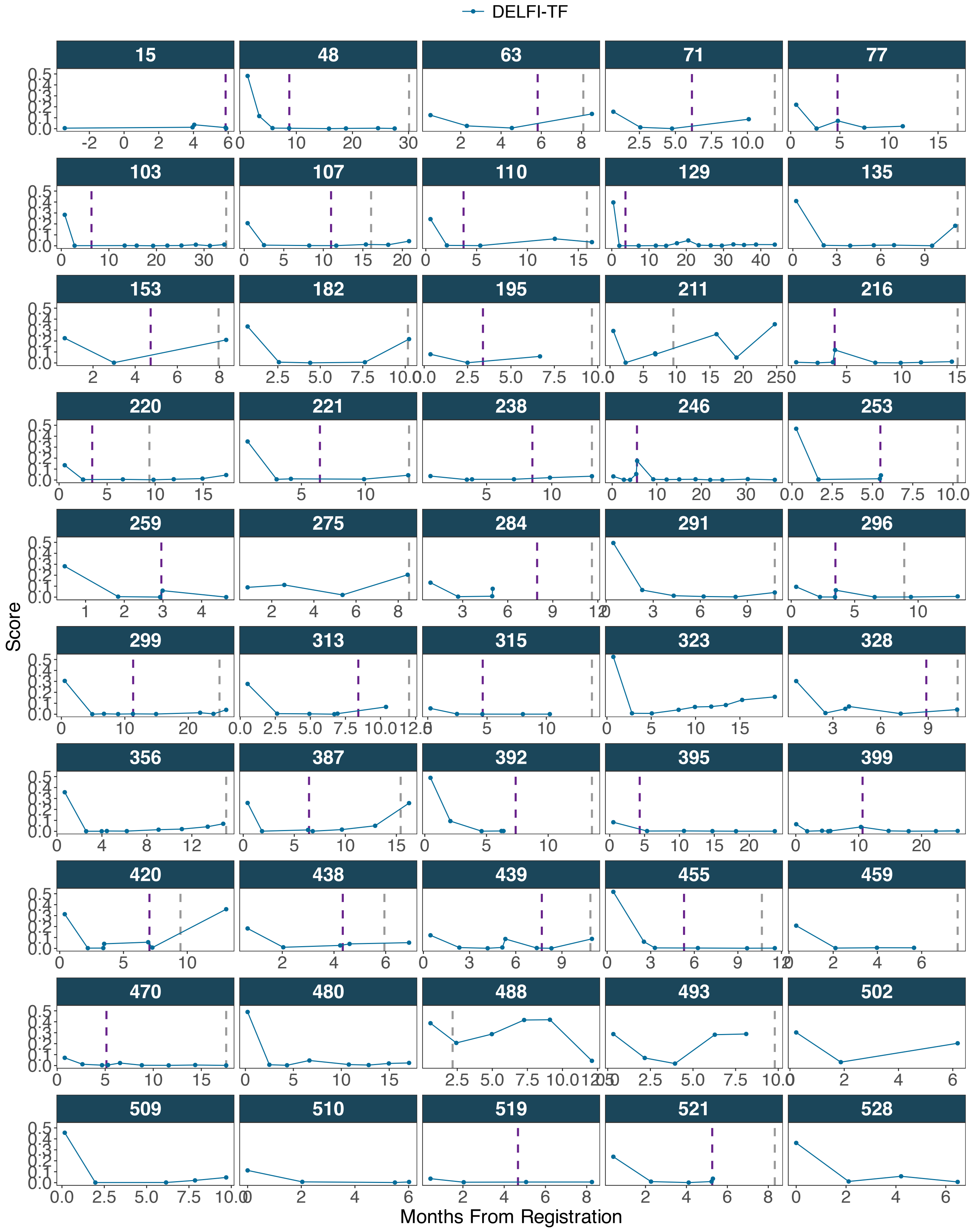

Supplementary Fig. 10. DELFI-TF dynamics in CAIRO5 patients in the wild type arm. Longitudinal DELFI-TF dynamics in patients with *RAS*/*BRAF* wild-type tumors. Purple vertical dashed lines represent surgical time points. Gray vertical dashed lines represent disease progression documentation by RECIST1.1.

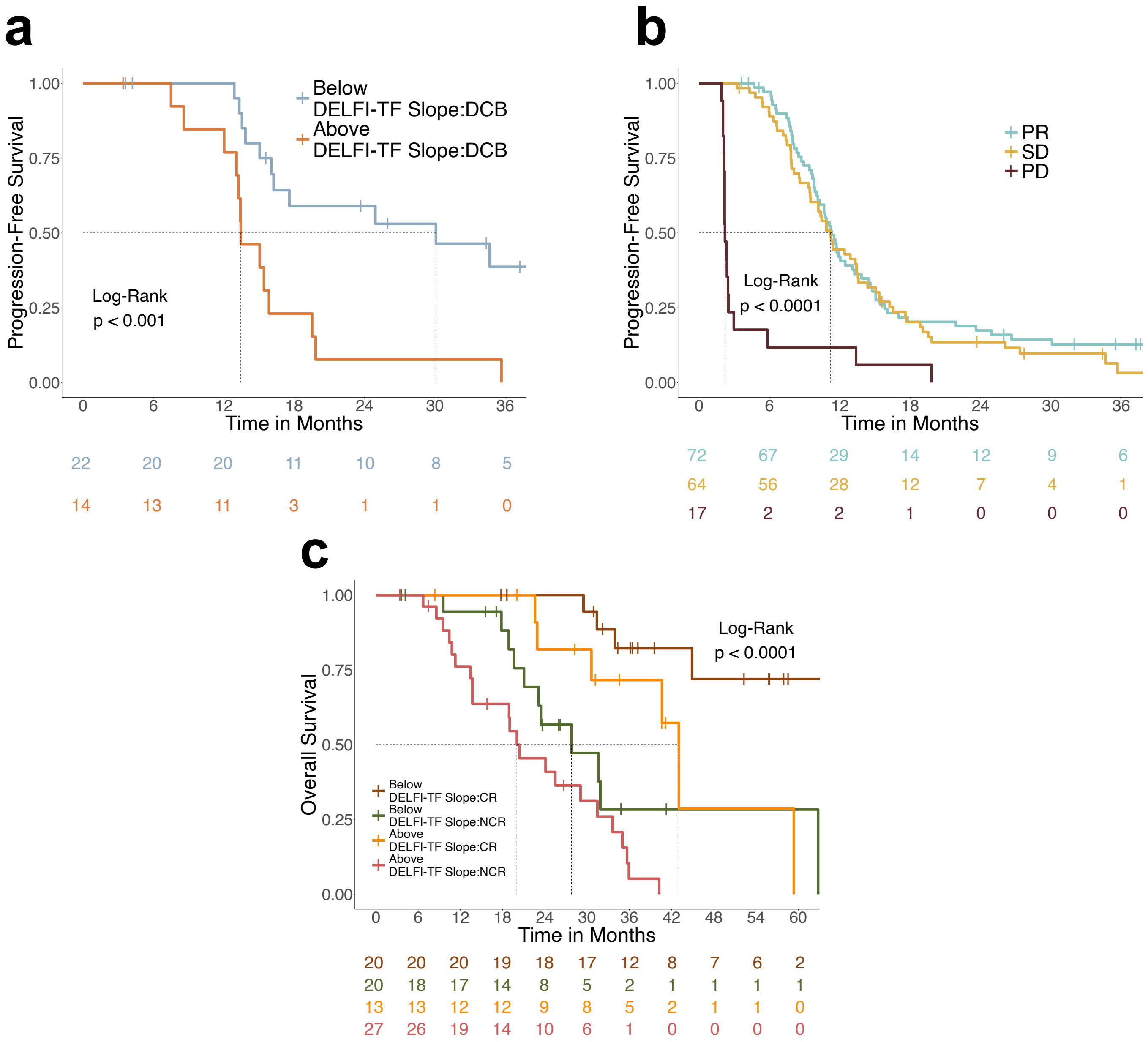

Supplementary Fig. 11. Imaging and plasma biomarkers for survival outcomes in metastatic colorectal cancer patients. a, Kaplan-Meier curves for progression-free survival (PFS) among 36 patients who experienced imaging response or stable disease that lasted longer than 12 months (durable clinical benefit) according to DELFI-TF slopes below (blue) or above (orange) the median in the overall study population (n=80) (*p=7.20e-04*, Log-Rank). **b**, Kaplan-Meier curves for PFS according to imaging response assessed by RECIST 1.1 show a significant survival difference with patients with progressive disease (PD) (red) and patients with partial response (PR) (blue) or stable disease (SD) (orange). **c**, Kaplan-Meier curves for overall survival (OS) according to surgical status and DELFI-TF slope among patients with at least one blood draw within 60 days of disease progression (n = 80) (*p=8.90e-09*, Log-Rank).
