# Supplementary material for: Cancer treatment monitoring using cell-free DNA fragmentomes
Source: Nat Commun. 2024 Oct 21;15:8801. doi: 10.1038/s41467-024-53017-7 (PMC11493959; doi:10.1038/s41467-024-53017-7)
Supplement: Supplementary file 3 — Reporting Summary [file 41467_2024_53017_MOESM3_ESM.pdf]

nature portfolio

Corresponding author(s): Remond J.A. Fijneman, Alessandro Leal

Last updated by author(s): Aug 13, 2024

Reporting Summary

Nature Portfolio wishes to improve the reproducibility of the work that we publish. This form provides structure for consistency and transparency in reporting. For further information on Nature Portfolio policies, see our Editorial Policies and the Editorial Policy Checklist.

Statistics

For all statistical analyses, confirm that the following items are present in the figure legend, table legend, main text, or Methods section.

n/a	Confirmed	
☐	☒	The exact sample size (*n*) for each experimental group/condition, given as a discrete number and unit of measurement
☐	☒	A statement on whether measurements were taken from distinct samples or whether the same sample was measured repeatedly
☐	☒	The statistical test(s) used AND whether they are one- or two-sided<br>*Only common tests should be described solely by name; describe more complex techniques in the Methods section.*
☐	☒	A description of all covariates tested
☐	☒	A description of any assumptions or corrections, such as tests of normality and adjustment for multiple comparisons
☐	☒	A full description of the statistical parameters including central tendency (e.g. means) or other basic estimates (e.g. regression coefficient) AND variation (e.g. standard deviation) or associated estimates of uncertainty (e.g. confidence intervals)
☐	☒	For null hypothesis testing, the test statistic (e.g. *F*, *t*, *r*) with confidence intervals, effect sizes, degrees of freedom and *P* value noted<br>*Give P values as exact values whenever suitable.*
☒	☐	For Bayesian analysis, information on the choice of priors and Markov chain Monte Carlo settings
☒	☐	For hierarchical and complex designs, identification of the appropriate level for tests and full reporting of outcomes
☐	☒	Estimates of effect sizes (e.g. Cohen's *d*, Pearson's *r*), indicating how they were calculated

Our web collection on statistics for biologists contains articles on many of the points above.

Software and code

Policy information about availability of computer code

Data collection	Plasma samples were collected from mCRC patients participating in the CAIRO5 clinical trial (NCT02162563). Sequence data from cfDNA was processed using fastp (0.20.1), samtools (1.13), bowtie2 (2.4.2). Picard (3.1.0) and Bedtools (2.26.0)
Data analysis	Data analysis was performed using R Statistical Software (version 4.3.3 Foundation for Statistical Computing, Vienna, Austria). Analysis is publicly available at https://github.com/delfidiagnostics/CAIRO5_Public. All package versions and supplemental information needed to recreate the analysis are available in supplemental materials or the public github repository. DELFI-TF dynamics and FFPE copy number analysis were performed using Python (3.9.13).

For manuscripts utilizing custom algorithms or software that are central to the research but not yet described in published literature, software must be made available to editors and reviewers. We strongly encourage code deposition in a community repository (e.g. GitHub). See the Nature Portfolio guidelines for submitting code & software for further information.

Data

Policy information about availability of data

All manuscripts must include a data availability statement. This statement should provide the following information, where applicable:

- Accession codes, unique identifiers, or web links for publicly available datasets
- A description of any restrictions on data availability
- For clinical datasets or third party data, please ensure that the statement adheres to our policy

De-identified data has been made available as part of the supplemental tables associated with this manuscript. Raw sequencing data have been deposited in EGA

under accession codes (EGAS00001006695, EGAS00001005340).

Scripts for reproducing tables and figures in the manuscript are available in the following GitHub repository (https://github.com/delfidiagnostics/CAIRO5_public).

Research involving human participants, their data, or biological material

Policy information about studies with human participants or human data. See also policy information about sex, gender (identity/presentation), and sexual orientation and race, ethnicity and racism.

Reporting on sex and gender	Both male and female patients were eligible for enrollment. The number of male and female patients randomized in this study has been previously reported. Huiskens, J. et al. Treatment strategies in colorectal cancer patients with initially unresectable liver-only metastases, a study protocol of the randomised phase 3 CAIRO5 study of the Dutch Colorectal Cancer Group (DCCG). BMC Cancer 15, 365 (2015). Allosomes were excluded from genomic data and there are no analysis based on sex and gender generated in this manuscript.
Reporting on race, ethnicity, or other socially relevant groupings	The number of patients by geographic region randomized in this study has been previously reported. Huiskens, J. et al. Treatment strategies in colorectal cancer patients with initially unresectable liver-only metastases, a study protocol of the randomised phase 3 CAIRO5 study of the Dutch Colorectal Cancer Group (DCCG). BMC Cancer 15, 365 (2015). There are no analysis based on race or ethnicity reported in this manuscript
Population characteristics	The population characteristics has been previously reported. Huiskens, J. et al. Treatment strategies in colorectal cancer patients with initially unresectable liver-only metastases, a study protocol of the randomised phase 3 CAIRO5 study of the Dutch Colorectal Cancer Group (DCCG). BMC Cancer 15, 365 (2015). Briefly, CAIRO5 enrolled patients were treated with doublet chemotherapy (FOLFOX or FOLFIRI) and bevacizumab with at least one blood draw prior to and after treatment initiation between March 2015 and November 2020 were included in the present study.
Recruitment	Patients with treatment-naïve non-operable liver-only mCRC enrolled in the CAIRO5 phase III trial. Recruitment of the CAIRO5 clinical trial was previously described: Huiskens, J. et al. Treatment strategies in colorectal cancer patients with initially unresectable liver-only metastases, a study protocol of the randomised phase 3 CAIRO5 study of the Dutch Colorectal Cancer Group (DCCG). BMC Cancer 15, 365 (2015).
Ethics oversight	The study was done in accordance with the standards of Good Clinical Practice and the Declaration of Helsinki. The study was approved by the medical ethical committee of the Amsterdam University Medical Centre, Amsterdam, The Netherlands. Data monitoring was done by The Netherlands Comprehensive Cancer Organisation. A data and safety monitoring board assessed all serious adverse events and data. All patients provided written informed consent to study procedures before enrollment.

Note that full information on the approval of the study protocol must also be provided in the manuscript.

Field-specific reporting

Please select the one below that is the best fit for your research. If you are not sure, read the appropriate sections before making your selection.

☒ Life sciences ☐ Behavioural & social sciences ☐ Ecological, evolutionary & environmental sciences

For a reference copy of the document with all sections, see nature.com/documents/nr-reporting-summary-flat.pdf

Life sciences study design

All studies must disclose on these points even when the disclosure is negative.

Sample size	This study is comprised of 689 samples across 2 arms (MT n=309; WT n=380). These samples were collected as part of a prospective phase III clinical trial. All patients treated with doublet chemotherapy and bevacizumab with at least one blood draw prior to and after treatment initiation between March 2015 and November 2020 were included in our study.
Data exclusions	No data were excluded from analysis other than sequencing data which failed library prep due to technical limitations.
Replication	Core findings from this study were verified in either the WT arm of the clinical trial or in an independent lung cancer validation cohort. Analyses scripts are publicly available and formated in such a way as to make the analysis reproducible.
Randomization	Patients randomization was previously described: Huiskens, J. et al. Treatment strategies in colorectal cancer patients with initially unresectable liver-only metastases, a study protocol of the randomised phase 3 CAIRO5 study of the Dutch Colorectal Cancer Group (DCCG). BMC Cancer 15, 365 (2015). Briefly, patients with RAS and BRAF wildtype and left-sided primary tumors will be randomised between FOLFOX or FOLFIRI plus either bevacizumab or panitumumab. The choice between FOLFOX or FOLFIRI is to the discretion of the local investigator, however, the treatment is restricted to regimens that are specified in the protocol. Patients with RAS or BRAF mutated and/or right-sided primary tumors will be randomized between FOLFOX/ FOLFIRI (investigator choice) plus bevacizumab or 5FU, irinotecan, oxaliplatin (FOLFOXIRI) plus bevacizumab.

Samples within this cohort which originated from the same individual had libraries constructed in the same batch in order to limit intra-sample batch effects. In addition inter and intra batch controls were used. We do not expect any residual batch effect to have an impact on the findings in this work.

Blinding	At the patient level, blinding procedures between participants and investigators is not applicable. At the sample level, an independent validation cohort was blindly evaluated for for circulating tumor fractions measured by the DELFI-TF assay.

Reporting for specific materials, systems and methods

We require information from authors about some types of materials, experimental systems and methods used in many studies. Here, indicate whether each material, system or method listed is relevant to your study. If you are not sure if a list item applies to your research, read the appropriate section before selecting a response.

Materials & experimental systems

n/a	Involved in the study
☒	☐ Antibodies
☒	☐ Eukaryotic cell lines
☒	☐ Palaeontology and archaeology
☒	☐ Animals and other organisms
☐	☒ Clinical data
☒	☐ Dual use research of concern
☒	☐ Plants

Methods

n/a	Involved in the study
☒	☐ ChIP-seq
☒	☐ Flow cytometry
☒	☐ MRI-based neuroimaging

Clinical data

Policy information about clinical studies

All manuscripts should comply with the ICMJEguidelines for publication of clinical research and a completedCONSORT checklist must be included with all submissions.

Clinical trial registration	NCT02162563
Study protocol	The present study is a retrospective analysis of liquid biopsies collected from a homogenous group of patients with mCRC participating in the prospective CAIRO5 clinical trial (NCT02162563). The phase III randomized CAIRO5 trial investigates the optimal first-line systemic therapy for patients with histologically proven CRC with isolated, previously untreated, initially unresectable liver metastases. Patients treated with doublet chemotherapy (FOLFOX or FOLFIRI) and bevacizumab with at least one blood draw prior to and after treatment initiation between March 2015 and November 2020 were included in the present study. All patients were considered unresectable at inclusion, i.e. R0-resection could not be achieved in one procedure with one surgical intervention. Upon treatment with doublet chemotherapy and bevacizumab, patients were evaluated every two months by an expert panel of liver surgeons and abdominal radiologists for the possibility of local treatment of colorectal liver metastases following current clinical practice. Clinical follow-up was performed according to the standard of care, including a clinical review every three months and CT imaging and serum CEA every six months. When the liver metastases stayed unresectable, chemotherapy was continued without the targeted agent for the total duration of pre- and post-operative treatment of six months, and patients were continuously evaluated until the progression of the disease by serum CEA and CT imaging every two months. Follow-up was recorded until September 1, 2021. The trial was approved by a medical ethical committee, performed according to the Declaration of Helsinki, and patients signed written informed consent for study participation and blood collection for translational research.
Data collection	From July 2014 till August 2022 samples were collected from patients enrolled in the CAIRO5 clinical trial. Patients selection, randomization and data collection was previously described: Huiskens, J. et al. Treatment strategies in colorectal cancer patients with initially unresectable liver-only metastases, a study protocol of the randomised phase 3 CAIRO5 study of the Dutch Colorectal Cancer Group (DCCG). BMC Cancer 15, 365 (2015).
Outcomes	The CAIRO5 clinical trial outcomes were previously described: Bond MJG, et al. First-line systemic treatment strategies in patients with initially unresectable colorectal cancer liver metastases (CAIRO5): an open-label, multicentre, randomised, controlled, phase 3 study from the Dutch Colorectal Cancer Group. Lancet Oncol. 2023;24(7):757-771. doi:10.1016/S1470-2045(23)00219-X<br>PFS : Time from registration until progression or death whichever comes first. R0/1 secondary resection rate. Median overall survival. Response rate R0/1 secondary resection rate in each of the 4 study arms upon neoadjuvant treatment with chemotherapy plus targeted therapy. Pathological complete response rate (pCR)

Plants

Seed stocks	*Report on the source of all seed stocks or other plant material used. If applicable, state the seed stock centre and catalogue number. If plant specimens were collected from the field, describe the collection location, date and sampling procedures.*
Novel plant genotypes	*Describe the methods by which all novel plant genotypes were produced. This includes those generated by transgenic approaches, gene editing, chemical/radiation-based mutagenesis and hybridization. For transgenic lines, describe the transformation method, the number of independent lines analyzed and the generation upon which experiments were performed. For gene-edited lines, describe the editor used, the endogenous sequence targeted for editing, the targeting guide RNA sequence (if applicable) and how the editor was applied.*
Authentication	*Describe any authentication procedures for each seed stock used or novel genotype generated. Describe any experiments used to assess the effect of a mutation and, where applicable, how potential secondary effects (e.g. second site T-DNA insertions, mosiacism, off-target gene editing) were examined.*
