# Supplementary material for: Cancer treatment monitoring using cell-free DNA fragmentomes
Source: Nat Commun. 2024 Oct 21;15:8801. doi: 10.1038/s41467-024-53017-7 (PMC11493959; doi:10.1038/s41467-024-53017-7)
Supplement: Supplementary file 4 — Description of Additional Supplementary Files [file 41467_2024_53017_MOESM4_ESM.pdf]

Supplementary Data

Supplementary Data 1 – Summary of clinical and demographic characteristics of patients in CAIRO5 trial

Supplementary Data 2 – Summary of blood sample analysis of patients in CAIRO5 trial

Supplementary Data 3 – Summary of cfDNA WGS statistics

Supplementary Data 4 – Summary of blood sample analyses of individuals without cancer in control cohort

Supplementary Data 5 – Summary of copy number alterations analyzed

Supplementary Data 6 – Summary of blood sample analyses of patients with lung cancer in validation cohort

Supplementary Data 7 – RECIST 1.1 sum of the longest diameters

Supplementary Data 8 – DELFI-TF slopes
